# Stage-Regulated GFP Expression in *Trypanosoma cruzi*: Applications from Host-Parasite Interactions to Drug Screening

**DOI:** 10.1371/journal.pone.0067441

**Published:** 2013-06-20

**Authors:** Rafael Luis Kessler, Daniela Fiori Gradia, Rita de Cássia Pontello Rampazzo, Édio Elígio Lourenço, Nilson José Fidêncio, Lauro Manhaes, Christian Macagnan Probst, Andréa Rodrigues Ávila, Stenio Perdigão Fragoso

**Affiliations:** Instituto Carlos Chagas, FIOCRUZ, Curitiba, Paraná, Brazil; Instituto Butantan, Laboratório Especial de Toxinologia Aplicada, Brazil

## Abstract

*Trypanosoma cruzi* is the etiological agent of Chagas disease, an illness that affects about 10 million people, mostly in South America, for which there is no effective treatment or vaccine. In this context, transgenic parasites expressing reporter genes are interesting tools for investigating parasite biology and host-parasite interactions, with a view to developing new strategies for disease prevention and treatment. We describe here the construction of a stably transfected fluorescent *T. cruzi* clone in which the GFP gene is integrated into the chromosome carrying the ribosomal cistron in *T. cruzi* Dm28c. This fluorescent *T. cruzi* produces detectable amounts of GFP only at replicative stages (epimastigote and amastigote), consistent with the larger amounts of GFP mRNA detected in these forms than in the non replicative trypomastigote stages. The fluorescence signal was also strongly correlated with the total number of parasites in *T. cruzi* cultures, providing a simple and rapid means of determining the growth inhibitory dose of anti-*T.cruzi* drugs in epimastigotes, by fluorometric microplate screening, and in amastigotes, by the flow cytometric quantification of *T. cruzi*-infected Vero cells. This fluorescent *T. cruzi* clone is, thus, an interesting tool for unbiased detection of the proliferating stages of the parasite, with multiple applications in the genetic analysis of *T. cruzi*, including analyses of host-parasite interactions, gene expression regulation and drug development.

## Introduction

*Trypanosoma cruzi* is a hemoflagellate protozoan parasite and the etiological agent of American trypanosomiasis, also known as Chagas disease. About 10 million people are thought to be infected with this parasite, mostly in Central and South America [1]. This parasite causes cardiac and/or gastrointestinal disorders, imposing a large economic and social burden on countries in which it is endemic [2]. It has a complex life cycle, comprising four distinct morphological forms and alternation between two phylogenetically distant hosts: the insect vector (which houses the epimastigote and metacyclic trypomastigote forms) and the mammalian host (housing the amastigote and bloodstream trypomastigote forms) (reviewed in [3], [4]). Epimastigotes and amastigotes are the replicative forms, whereas metacyclic and bloodstream trypomastigotes are infectious, non-replicative forms.

*T. cruzi* and other trypanosomatids are not only important human parasites, they also present atypical molecular mechanisms for the regulation of gene expression, including polycistronic transcription. In this process, the individual mature mRNAs are created by the processing of pre-mRNAs through *trans*-splicing and polyadenylation (reviewed in [5]). No canonical RNA pol II promoters have been described and no clear case in which the initiation of transcription is regulated for this polymerase has yet been reported. Moreover, genes present in the same polycistronic unit and transcribed as a single pre-mRNA have different patterns of expression. Thus, gene expression in trypanosomatids is controlled largely by posttranscriptional events, modifying mRNA stability and translation (reviewed in [6], [7]).

The polycistronic processing of mRNA by *trans*-splicing makes it possible for transcription to be mediated by other RNA polymerases (reviewed in [8]). Thus, RNA pol I, which transcribes the pre-rRNA gene cluster, can mediate the expression of exogenously introduced protein-coding genes under the control of the strong ribosomal promoter, provided that the coding region of interest is flanked by intergenic regions (IRs) containing sequence elements mediating *trans*-splicing and polyadenylation. Several studies have made use of constructs including the rRNA promoter, to induce high levels of exogenous gene transcription in *T. cruzi*, in both transient [9], [10], [11], [12] and stable transfection conditions [11], [13], [14], [15].

The development of transgenic parasites expressing reporter genes has increased our knowledge of host-parasite interactions. Fluorescent proteins (FPs) have proved particularly useful in this respect [16]. FPs are intrinsically fluorescent, so their detection requires no particular preparation, simplifying reporter quantification in standard fluorescence systems based on microplate readers, FACS or fluorescence microscopy. The heterologous expression of green fluorescent protein (GFP) paved the way for new applications of FPs and has revolutionized imaging in cell biology [17]. GFP has already been expressed in several *Leishmania* [18], [19], [20], *Trypanosoma* [13], [21], [22], [23], *Toxoplasma* [24] and *Plasmodium* species [25], [26]. The construction of parasite cell lines producing fluorescent proteins has wider applications, in studies of subjects ranging from pathogenesis to protein function [12], [16], [27].

We describe here the construction of a fluorescent *T. cruzi* clone expressing the GFP gene integrated into the 18S ribosomal RNA locus. Unlike other fluorescent *T. cruzi* cell lines [22], [23], this particular clone produces GFP in detectable amounts only in replicative stages of the parasite (epimastigotes and amastigotes). This tool is therefore useful for the unbiased detection of proliferating parasites, and has multiple possible applications in the genetic analysis of *T. cruzi*, including analyses of host-parasite interactions, gene expression regulation, and in drug development.

## Materials and Methods

### Trypanosoma Cruzi Cultures

#### Parasite culture

*T. cruzi* Dm28c epimastigotes [28] were cultured at 28°C in LIT medium supplemented with 10% fetal bovine serum. The culture was initiated by adding 1×10^6^ cells ml^−1^ and the parasites were harvested when the culture reached a cell density of 2 to 3×10^7^ cells ml^−1^ (exponential growth-phase parasites).

#### In vitro metacyclogenesis

We obtained metacyclic trypomastigotes, by allowing *T. cruzi* epimastigotes to differentiate under chemically defined conditions (TAU3AAG medium), as previously described [28], [29]. Briefly, epimastigotes in the late exponential growth phase were harvested from LIT medium by centrifugation and subjected to nutritional stress for 2 h in triatomine artificial urine (TAU: 190 mM NaCl, 17 mM KCl, 2 mM MgCl_2_, 2 mM CaCl_2_, 8 mM sodium phosphate buffer, pH 6.0) at a density of 5×10^8^ cells ml^−1^. Stressed parasites were used to inoculate cell culture flasks containing TAU3AAG (TAU supplemented with 50 mM sodium glutamate, 10 mM L-proline, 2 mM sodium aspartate, 10 mM glucose), at a density of 5×10^6^ cells ml^−1^, at 28°C. Metacyclic trypomastigotes were purified by DEAE-51 chromatography from the TAU3AAG culture supernatant after 72 h of incubation.

#### Infection of cultured mammalian cells

Mammalian cell invasion experiments were performed as previously described [30]. Vero cells were maintained in RPMI medium supplemented with 5% bovine fetal serum (Invitrogen, Carlsbad, CA, USA), 100 IU/ml penicillin, 10 µg/ml streptomycin and 2 mM glutamine, at 37°C, under an atmosphere containing 5% CO_2_. Cell-derived trypomastigotes were obtained by infecting Vero cell cultures at 50 to 70% confluence with metacyclic trypomastigotes, with 100 parasites per host cell. After 24 h, the cells were washed once with RPMI to remove non adherent metacyclic forms and fresh medium was added to the culture flask. Cell-derived trypomastigotes were released into the supernatant 4 to 5 days after infection and were harvested by centrifugation at 5,000×*g* for 10 min. The cell-derived trypomastigotes were used to infect new Vero cultures, with 10 parasites added per host cell. In these conditions, the production of amastigotes and trypomastigotes peaked after two and four days of infection, respectively.

### Plasmid Construction to Ensure Integration of the GFP Gene into the Ribosomal Locus

Two regions of the 18S rDNA of *T. cruzi* (GenBank accession number AF245382.1), from nucleotides 50 to 840 (18SA, 790 bp) and from nucleotides 1130 to 1942 (18SB, 812 bp), were amplified by PCR with the primer pairs 18SAF+18SAR and 18SBF+18SBR (table 1), respectively, and inserted into the pBluescript (SK+) II plasmid (Stratagene, La Jolla, CA, USA). The 18SA fragment was inserted between the *Sal*I and *Hin*dIII sites, whereas the 18SB fragment was inserted between the *Sac*I and *Sac*II sites. The recombinant plasmid was named pKS/18S. The neomycin phosphotransferase (NEO) gene encoding resistance to G418 was used as a selectable marker and was amplified by PCR with the NEOF and NEOR primers (table 1). The amplicon was inserted into the pKS/18S plasmid, between the *Xba*I and *Sac*II sites. This recombinant plasmid was named pKS/18S/NEO.

**Table 1:** Primers used for plasmid construction.

Sequences	Forward primers (5′–3′)	Reverse primers (5′–3′)
18SA	ATC**GTCGAC**CATGCATGCCTCAGAATCTTCACCTGT	CTT**AAGCTT**CGCCCTCTGGCATGCATGACAT
18SB	GTTC**GAGCTC**ATGTGTCATGCCTTCCCTCAACT	GTTC**CCGCGG**ACATTGAGGAGCATCACAGACCT
NEO	TCGT**TCTAGA**TGATTGAACAAGATGGATTG	AGAG**CCGCGG**TCAGAAGAACTCGTCAA
GFP	GGG**GGATCC**ATGGTGAGCAAGGGCGAGGAGC	GGTGGAT**GGATCC**CCCGCGGTACTTACGACTGC
IRA	GAGGAG**AAGCTT**ACCTGTAAATAGACCAATG	ATTGG**GATATC**GAAAGAAGAAAGAGGCGAGTAGA
IRB	AGGAG**GGATCC**AAGTGTCAAATAGACCAA	CATTGG**TCTAGA**GAAAGAAGAAAGAGGCGAG
IRC	AGGAG**CCGCGG**ACGTGTCAAATAGACCAATG	CATTGG**CCGCGG**GAAAGAAGAAAGAGGCGAG

Table 1 legend: NEO, neomycin phosphotransferase gene; IRA: intergenic region A; IRB: intergenic region B; IRC: intergenic region C. The restriction enzyme cleavage sites are shown in bold.

The intergenic region (387 bp) between the alpha and beta tubulin genes from the insect trypanosomatid *Strigomonas culicis* (formerly *Blastocrithidia culicis* [31]) (GenBank accession number JX045653) was amplified by PCR with the following three pairs of primers: IR1+IR2 (IRA), IR3+IR4 (IRB) and IR5+IR6 (IRC) (table 1). The IRA fragment was inserted into the pKS/18S/NEO plasmid, between the *Hin*dIII and *Eco*RV sites. The resulting recombinant plasmid was used to insert the IRB region between the *Bam*HI and *Xba*I sites. The IRC fragment was then inserted into the *Sac*II site of the resulting plasmid, to obtain the expression vector, pBEX.

The GFP gene was amplified from pEGFP-C3 (Clontech, Palo Alto, CA, USA) with the primers GFPF and GFPR (table 1) and inserted into the *Bam*HI site of pBEX. The resulting plasmid was named pBEX/GFP and used to transfect epimastigote forms of *T. cruzi*.

### Parasite Transfection

Epimastigote forms of *T. cruzi* were grown to a density of approximately 3×10^7^ cells ml^−1^. They were then harvested by centrifugation at 4,000×*g* for 5 min at room temperature, washed once in phosphate-buffered saline (PBS) and resuspended in 0.4 ml of electroporation buffer (140 mM NaCl, 25 mM HEPES, 0.74 mM Na_2_HPO_4_, pH 7.5) at a density of 1×10^8^ cells ml^−1^. Cells were then transferred to a 0.2 cm path-length cuvette and 50 µg of pBEX/GFP plasmid was added. The mixture was placed on ice for 10 min and then subjected to 2 pulses of 450 V and 500 µF with a Gene Pulser II (Bio-Rad, Hercules, USA). The electroporated cells were then maintained on ice until their transfer to 10 ml of LIT medium supplemented with 10% fetal bovine serum, in which they were incubated at 28°C. After 24 h, 300 µg/ml G418 (Sigma, St. Louis,MO,USA) was added, to select for transfected parasites. Transfectants were cloned by serial dilution in 24-well plates. The insertion of the cassette into the ribosomal locus was analyzed by pulsed-field gel electrophoresis followed by Southern blot analysis.

### Separation of *T. cruzi* Chromosomal DNA by Pulsed-field Gel Electrophoresis (PFGE)

A transfectant clone of *T. cruzi* expressing the GFP gene was grown to late exponential growth phase. Cells were collected in PBS and mixed with an equal volume of 1% low-melting point agarose. We used approximately 1×10^7^ cells (100 ml) for each gel plug. The gel plugs were incubated in a solution containing 0.5 M EDTA (pH 8.0), 1% sodium lauryl sarcosinate (Sarkosyl) and 1 mg/ml proteinase K, at 50°C, for 48 h, and were stored at 4°C in 0.5 M EDTA (pH 8.0). Samples were run on the agarose gel and gel plugs containing wild-type epimastigotes were used as a control. Chromosomal bands were separated with a LKB Gene Navigator System (Pharmacia LKB, Uppsala, Sweden) and a hexagonal electrode array. PFGE was carried out in a 0.8% agarose gel, in 0.5×TBE (45 mM Tris; 45 mM boric acid; 1 mM EDTA, pH 8.3) at 10°C, with a program including five phases of homogeneous pulses (N/S, E/W), for 135 h, with an interpolation of 83 V. The pulse times were 90 s for phase 1 (run time 30 h), 200 s for phase 2 (30 h), 350 s for phase 3 (25 h), 500 s for phase 4 (25 h) and 800 s for phase 5 (25 h). Chromosomes from *Hansenula wingei* (Bio-Rad, Hercules, CA, USA) were used as molecular mass standards. The gel was stained with ethidium bromide and photographed with a molecular imaging system (Loccus Biotecnologia, Cotia, SP, Brazil). DNA bands were transferred to nylon membranes in 20×SSC and hybridized with the following probes: the GFP gene (complete coding sequence), a 589 bp fragment of rDNA 24S alpha (generated by PCR amplification with the following primers: 24SαF: AGCGAAAGACTCATCGAACCAC and 24SαR: CTCAGATGGACCAGCCTCTA) and the 18SA fragment (790 bp) from the pBEX construct. The probes were labeled with phosphorus-32 by nick translation, according to standard protocols [32].

### Immunoblotting

Cell lysates for the epimastigote and metacyclic trypomastigote forms of GFP-producing *T. cruzi* were subjected to SDS-PAGE in 15% polyacrylamide gels and the protein bands were transferred onto a nitrocellulose membrane (Hybond C, Amersham Biosciences, Buckinghamshire, UK) according to standard protocols [32]. Nonspecific binding sites were blocked by incubating the membrane for 30 min in 5% non-fat milk powder and 0.1% Tween-20 in PBS, pH 8.0. The membrane was then incubated for 1 h with a polyclonal antiserum raised against the recombinant protein GFP (1∶500 dilution) in blocking solution. The membrane was washed three times in TBS and incubated for 45 min with horseradish peroxidase-conjugated anti-rabbit IgG (Amersham Biosciences) diluted 1∶7,500 in blocking solution. Bound antibodies were detected with the ECL western blot analysis system (Amersham Biosciences), according to the manufacturer’s instructions.

### Flow Cytometry

Epimastigotes and purified metacyclic trypomastigotes (1×10^6^ cells) were harvested by centrifugation at 2,000×*g* for 5 min, washed with PBS and resuspended in 1 ml of PBS for the immediate quantification of GFP fluorescence by flow cytometry. For the analysis of intracellular amastigotes, Vero cells were released from the culture flasks by trypsin treatment and mechanically lysed by successive passages through a 25-gauge needle coupled to a 1 ml syringe. The released amastigotes were washed with PBS and resuspended in 1 ml of PBS for quantification by flow cytometry. Cell-derived trypomastigotes were obtained directly from the supernatant of infected Vero cells and were washed as described above. Flow cytometry analyses were carried out in a FACSCalibur (Becton-Dickinson, San Jose, USA) and GFP fluorescence was analyzed with an FL1-H detector (530/30 nm band-pass filter). We acquired a total of 20,000 events in the scatter regions previously shown to correspond to parasite cells. Data analysis was performed with FlowJo software (Treestar software).

Cell viability was analyzed by harvesting 1×10^6^ epimastigotes by centrifugation, as described above, and resuspending them in 5 µg ml^−1^ propidium iodide (PI) in PBS. The cells were incubated for 15 minutes at 28°C and were then immediately quantified, without washing, with an FL2-H detector (585/42 nm band-pass filter). Positively stained cells were considered to be dead.

Cell cycle analysis was performed by staining the DNA with PI. Epimastigotes (1×10^6^ cells) were harvested, washed in PBS and fixed by incubation with 2% formaldehyde in PBS for 1 min. Cells were washed once in PBS and resuspended in 500 µl of PBS, to which we added an equal volume of DNA staining solution (3.4 mM Tris-HCl, 75 µM PI, 0.1% NP40 detergent, 10 mM NaCl and 700 U/l RNase A). The cell suspension was kept on ice until quantification by flow cytometry. PI fluorescence was quantified with a 610/20 nm band-pass filter and GFP was quantified with a 530/30 nm band-pass filter, in aFACS AriaII cytometer (Becton-Dickinson, San Jose, USA). Wild-type and PBEX parasites were used for detector signal compensation. We analyzed the data for 610/20 nm-width×610/20 nm-area gated cells to exclude debris and doublets. The Dean-Jett-Fox algorithm of FlowJo was used to estimate the percentages of cells in the G0/G1, S and G2/M phases of the cell cycle.

### Fluorescence Confocal Microscopy

For the analysis of GFP fluorescence in epimastigotes and purified metacyclic trypomastigotes, cell suspensions were washed with PBS and fixed by incubation with 4% paraformaldehyde in PBS for 10 min. Parasites were washed with PBS and allowed to adhere to 0.1% poly-L-lysine-coated slides for 15 min. They were then washed again with PBS to remove non adherent cells. DNA was stained by incubation for 5 min with 20 µl of 2 µg/µl Hoechst 33342 (Invitrogen) in PBS and were then washed five times in PBS. We then added 10 µl of *n*-propyl gallate (anti-fading solution) and sealed the slides with a coverslip and sealer (clear nail varnish).

For the analysis of amastigotes and cell-derived trypomastigotes, Vero cells were seeded on 13-mm round coverslips in 24-well plates and were infected with the parasite directly on the coverslip, as previously described. After 2 and 4 days of infection, corresponding to the periods at which amastigotes and trypomastigotes, respectively, predominated in the Vero cell culture, the coverslips were washed with prewarmed PBS and fixed by incubation with 4% paraformaldehyde for 10 min. The coverslips were washed with PBS and the DNA was stained as described above. They were washed three times with PBS and placed over 10 µl of *n*-propyl gallate on glass slides, which were then sealed with clear nail varnish.

The slides were analyzed in a Leica SP5 scanning confocal microscope and the images obtained were subsequently processed with Adobe Photoshop CS2 software, to improve contrast.

### RNA Purification and qPCR

Total RNA was extracted from epimastigote and metacyclic trypomastigote forms (1×10^9^ cells) with the RNeasy kit (Qiagen, Hilden, Germany), according to the manufacturer’s instructions, with an additional on-column DNase digestion step. RNA integrity was assessed with an Agilent 2100 Bioanalyzer and the RNA 6000 Nano LabChip kit, according to the manufacturer’s instructions. First-strand cDNA was synthesized and qPCR was carried out as previously described [12]. The first-strand cDNA was obtained by mixing 1 µg of total RNA and 1 µM oligo dT and incubating at 70°C for 10 min. We then mixed 4 µl of Improm-II buffer (Promega, Madison, USA), 3 mM MgCl_2_, 0.5 mM of each dNTP, 40 U RNaseOUT (Invitrogen) and 2 µl Improm-II reverse transcriptase (Promega) in a final volume of 20 µl and incubated the mixture for 2 h at 42°C. The product was then purified with Microcon(r) YM-30 (Millipore, Massachusetts, USA) and resuspended at a concentration of 2 ng µl^−1^ in water.

We used the relative standard curve method for qPCR analysis of GFP and NEO mRNA levels. Equal amounts of single-stranded cDNAs from epimastigotes and metacyclic trypomastigotes were mixed and five serial dilutions, containing 50 to 0.08 ng of cDNA, were used to generate standard curves for each pair of PCR primers. Each test PCR included 10 ng of template cDNA, 0.25 µmol of each oligonucleotide and SYBR(r) Green PCR Master Mix (Applied Biosystems, Foster City, USA). We used L9 ribosomal protein (forward: 5′-CCTTCACTGCCGTTCGTTGGTTTG-3′, reverse: 5′-ATGCGAGAGTGCCGTGTTGATGGT-3′) and H2B histone (forward: 5′-CGGTGGTGCGCGTCAACAAGAAGC-3, reverse: 5′-CCAGGTCCGCCGGCAGCACGAG-3′) primers as endogenous controls for normalization, and GFP (forward: 5′-TCTTCAAGGACGACGGCAAC-3, reverse: 5′-GGTCTAGACTTGTACAGCTCGTCCATG-3′) and NEO primers (forward: GGCTCGAGATGGGTAAGGAAAAGACTCAC, reverse: GGCTCGAGTTAGAAAAACTCATCGAGCATC) for target gene quantification. After initial incubations at 50°C for 5 min and 95°C for 10 min, the samples were subjected to 45 cycles of amplification, as follows: 95°C for 15 s, 60°C for 20 s and 72°C for 1 min. All standard curve point determinations and test reactions were performed in triplicate. The PCR runs were carried out in an Applied Biosystems 7500 Real-Time PCR System and data were acquired with Real-Time PCR System Detection Software v1.4 (Applied Biosystems).

### Epimastigote Fluorometric Assays

GFP-expressing epimastigotes were used to inoculate 96-well plates at various cell densities (in triplicate), in a final volume of 200 µl, and the corresponding fluorescence intensities were measured directly in LIT medium at room temperature with a microplate reader (Synergy H1 Hybrid Multi-Mode Microplate Reader, Biotek) and excitation/emission wavelengths of 488/528 nm, using LIT medium for the blank.

For drug testing, epimastigotes at an initial density of 1×10^7^ cells ml^−1^ were exposed to serial dilutions of benznidazole (BZ) (in quintuplicate) and the fluorescence intensity of treated cultures was measured after 24 h, with LIT plus BZ used as the blank. The IC_50_/24 h of BZ was obtained by nonlinear regression analysis, with a sigmoidal function in GraphPad Prism software (Version 5.00), after the plotting of log_2_ drug concentration against mean growth inhibition (fluorescence intensity of treated cells/untreated control). The standard deviation of the IC_50_/24 h was estimated by the best-fitted parameters of the sigmoid function.

### Flow Cytometry Assay for Anti-amastigote Activity

Vero cells were used to seed six-well plates, at a density of 2×10^5^ cells per well. The following day, cell-derived trypomastigotes were used to infect Vero cells at infection ratios of 1, 2, 4, 8, 16 and 32 trypomastigotes per host cell, in quadruplicate. After 4 h of interaction, non adherent trypomastigotes were removed by washing with PBS and fresh medium was added to the culture plates. After 48 hours of infection (peak of intracellular amastigote production), Vero cells were washed twice with PBS, fixed with methanol and stained with Giemsa stain (in duplicate). The plates were observed under an inverted microscope (400×magnification) and six noncontiguous, randomly selected fields were photographed per well. The percentage of cells infected and the number of amastigotes per host cell were determined with the Cell Counter Plugin of Image J (Mac Biophotonics Version). Amastigote growth was quantified with the microscopy infection index (IF_M_), obtained by multiplying the mean percentage infected cells by the mean number of amastigotes per infected cell.

In parallel, the infected host cells were analyzed by flow cytometry (in duplicate). Vero cells were detached from the wells of the plate, by adding 1 ml of 0.1% trypsin and incubating for 10 min, and then homogenizing the cells gently by passage through a Pasteur pipette. Trypsin was inactivated by adding 200 µl of fetal bovine serum and the cells were immediately analyzed by flow cytometry, using the FSCxSSC region corresponding to intact Vero cells. GFP fluorescence was quantified with a 530/30 nm band-pass filter, and GFP-positive Vero cells were considered to be infected. Amastigote growth was quantified by calculating the flow cytometry infection index (IF_FC_) as the mean percentage infected cells (GFP^+^) multiplied by the median fluorescence intensity of the GFP^+^ population.

For drug assays, Vero cells were infected with a 10∶1 ratio of trypomastigotes to cells, in six-well plates. Non adherent trypomastigotes were removed after 4 h, as described above. After 24 h of infection, Vero cell cultures were exposed to serial dilutions of BZ (200–0.195 µM) (in duplicate) for 24 h and amastigote growth inhibition was then analyzed by flow cytometry (at 48 h of infection). The IC_50_/24 h of BZ was obtained by nonlinear regression analysis in GraphPad Prism software (Version 5.00), after the plotting of log_2_ drug concentration against mean relative IF_FC_ (IF_FC_ treated/IF_FC_ non treated).

## Results

### Characterization of Transfected *T. cruzi* Cell Lines

**Figure 1:**
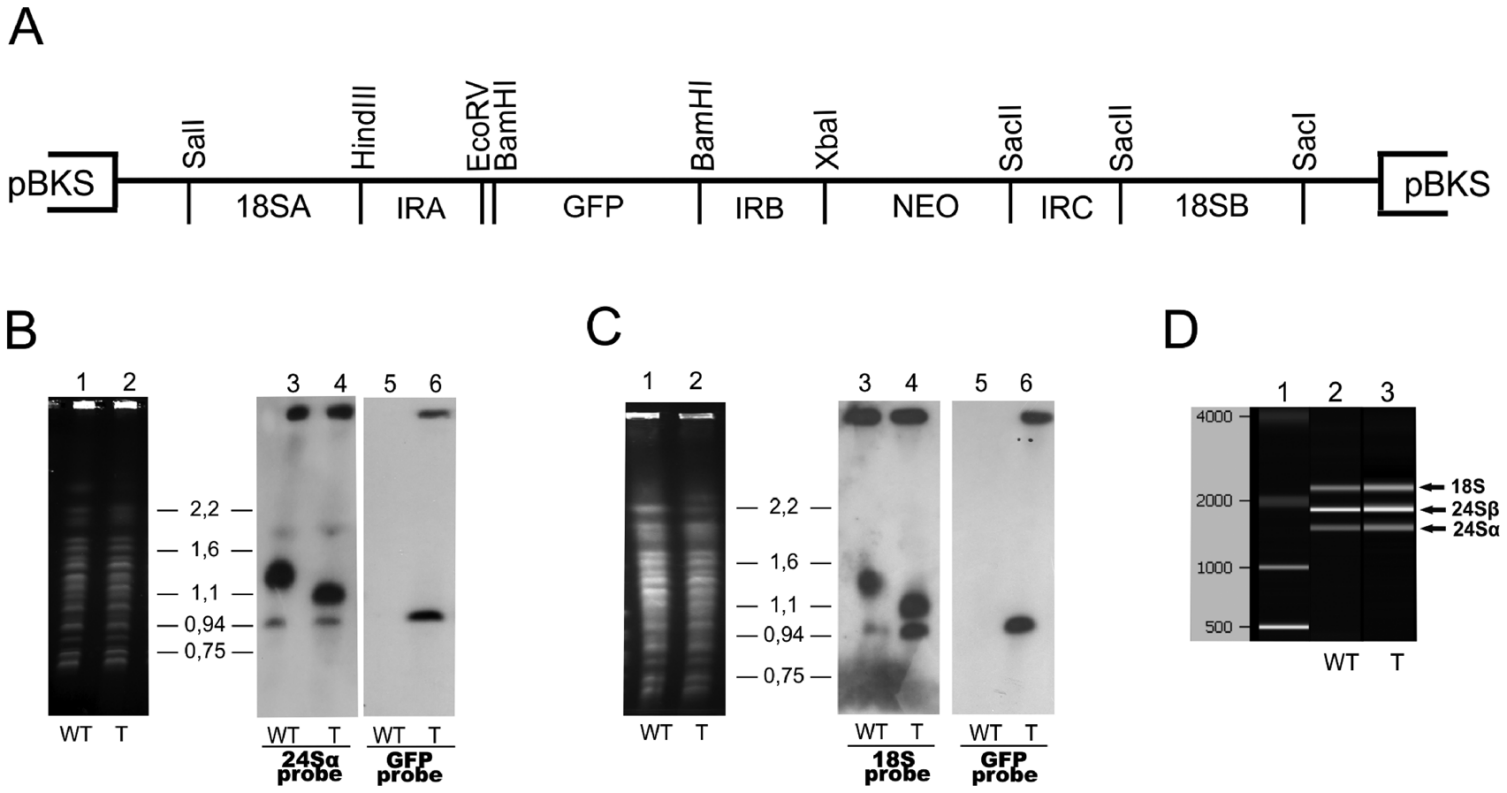
Integration of the green fluorescent protein (GFP) gene following parasite transfection. (A) Schematic representation of the pBEX/GFP construct. The expression vector has the *Trypanosoma cruzi* 18S ribosomal sequences flanking the intergenic regions between the alpha and beta tubulin genes that provides the spliced leader and polyadenylation sites for the GFP mRNA and the neomycin resistance gene (NeoR) used as a selectable marker. (B and C) Southern-blot analyses of transfected parasites. High-molecular weight DNA, isolated from wild-type (WT) epimastigotes of *T. cruzi* Dm28c (B1 and C1) and Dm28c transfected (T) with pBEX/GFP (fluorescent epimastigotes) (B2 and C2) were separated by PFGE and stained with ethidium bromide. The bands were transferred to nylon membranes and hybridized with [^32^P]-labeled probes corresponding to the 24S alpha rDNA (B3 and B4), 18S rDNA (C3 and C4) and GFP (B5, B6, C5 and C6) sequences. (D) Total RNA was isolated from wild-type epimastigotes and pBEX fluorescent epimastigotes and analyzed with an Agilent 2100 Bioanalyzer; data are displayed as a densitometry plot (gel-like image). In this analysis, the fluorescent parasites display a rRNA band pattern (D3) similar to that of the wild-type parasites (D2), suggesting that the mobility shift of the 1.4 Mbp chromosome did not affect the production of functional rRNA molecules. D1 =  molecular weight marker.

The pBEX/GFP vector was constructed to allow the integration of the GFP gene into the 18S ribosomal locus of *T. cruzi* (Figure 1A). The intergenic regions between the alpha and beta tubulin genes (IRA, IRB and IRC) were derived from *S. culicis* rather than *T. cruzi*. This strategy made it possible to use similar IRs (IRA, IRB, IRC) to achieve the correct processing of both NEO and GFP transcripts and to reduce the possibility of recombination events occurring at the tubulin loci of *T. cruzi*.

The integration of the construct into the parasite genome was demonstrated by pulsed field gel electrophoresis analysis and Southern blotting. The 18S and 24S alpha rDNA probes identified the 1.0 and 1.4 Mb chromosomes, respectively, which carry the ribosomal cistron in the *T. cruzi* Dm28c clone (Figure 1B and 1C). The 1.4 Mb band was more intensely labeled than the 1.0 Mb band, suggesting that the two chromosomes have different numbers of rDNA transcription units. On both blots, the GFP probe gave a hybridization pattern consistent with the 1.0 Mb chromosomal band (Figure 1B.6 and 1C.6), indicating that the GFP gene was integrated into a chromosome containing the minor cluster of ribosomal genes in the fluorescent *T. cruzi* clone. Surprisingly, the higher molecular weight band recognized by the ribosomal probes in the fluorescent *T. cruzi* clone was about 0.3 Mb smaller than that in the wild type, whereas the size of the chromosome into which the GFP gene was integrated was unaffected (Figure 1B.4 and 1C.4). An analysis of ribosomal RNA isolated from the wild-type parasite and the GFP-expressing *T. cruzi* clone showed that the two parasites had similar ribosomal RNA profiles (Figure 1D.2 and 1D.3), suggesting that decrease in the size of the larger chromosome carrying the ribosomal locus did not affect the synthesis of functional rRNAs in the transfectant. We have no plausible explanation for this variation of chromosome length as yet.

GFP production was observed during 52 weekly passages of axenic epimastigote cultures in the absence of the selective drug (G418). Thus, stable maintenance and expression of the GFP gene overcomes the problems of heterogeneous levels of gene expression in parasite populations due to differences in plasmid copy number and the instability of transgene expression in the absence of the selective drug [33].

### GFP Expression does not Affect Parasite Growth and Differentiation

**Figure 2:**
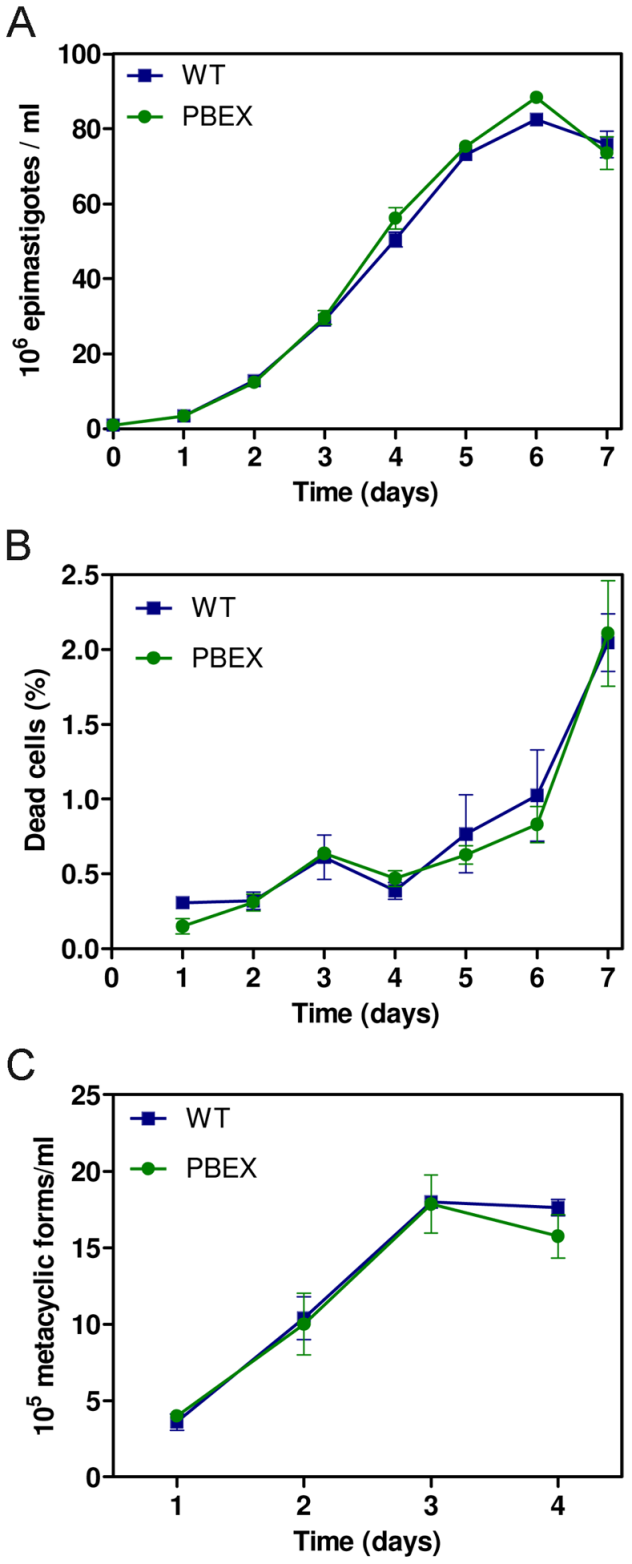
GFP expression does not affect the growth, viability and metacyclogenesis of *T. cruzi*. (A) Growth curves of wild-type (Dm28c) and pBEX/GFP cultured epimastigotes showing no significant difference in growth; cell density was determined with an electronic particle counter (Z2 Coulter Particle Count and Size Analyzer, Beckman Coulter®). (B) Cell viability analysis of epimastigote cultures by vital dye (propidium iodide - PI) staining and flow cytometry quantification of PI-positive cells. (C) Metacyclogenesis efficiency in wild-type and pBEX/GFP cultures; after metacyclogenesis induction (details in methods) the density of metacyclic forms in the culture supernatant was obtained daily, by direct counting in a Neubauer chamber. For all plots, each experimental point represents the mean and standard deviation of triplicate experiments.

We investigated whether GFP expression had any negative effects on epimastigote proliferation, viability and the ability to differentiate into metacyclic trypomastigotes. GFP-producing epimastigotes and wild-type parasites had similar growth rates, reaching the stationary growth phase after six days of culture (Figure 2A). Furthermore, flow cytometry evaluations of cell viability based on propidium iodide staining showed that only a small percentage of GFP-producing *T. cruzi* parasites in axenic cultures were non viable, this proportion being similar to that for wild-type parasites (Figure 2B).

The capacity of fluorescent epimastigotes to differentiate into metacyclic trypomastigotes was analyzed by *in vitro* metacyclogenesis. Metacyclic forms in the culture supernatant were quantified by daily differential counting after the induction of differentiation. The degree of differentiation of GFP-expressing *T. cruzi* epimastigotes into metacyclic forms was similar to that of wild-type parasites (Figure 2C). In addition, metacyclic and cell-derived trypomastigote forms of the GFP-producing *T. cruzi* clone infected Vero cells as efficiently as wild-type parasites, taking the same amount of time to complete the intracellular cycle as control parasites (data not shown).

### Only the Replicative Stages of the Parasite Expressed GFP

**Figure 3:**
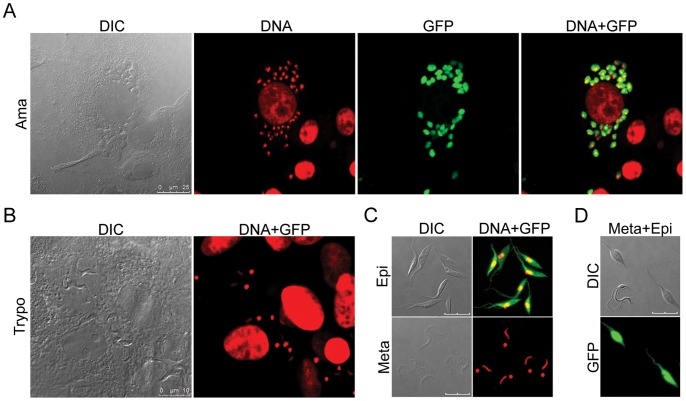
Fluorescence confocal microscopy assessing GFP expression in various stages of *T. cruzi* pBEX/GFP. (A) Amastigote-infected Vero cells after 48 h of trypomastigote infection. (B) Trypomastigotes produced after 4 days of Vero cell infection. (C) Epimastigotes in the exponential growth phase (upper photos) and purified metacyclic forms (lower photos). (D) Mixture of live epimastigotes and metacyclic forms, showing the absence of GFP fluorescence in trypomastigotes. DIC: differential interference contrast; DNA: staining of DNA with Hoechst 33342 dye (for better visualization in overlay images, Hoechst fluorescence has been artificially converted to red); GFP: fluorescence of GFP; DNA+GFP: overlay images of Hoechst and GFP fluorescence. Bars correspond to 10 µm, except in A (25 µm).

GFP expression was analyzed in all major stages of *T. cruzi*, by fluorescence confocal microscopy and flow cytometry. Microscopy showed that the replicative stages (epimastigotes and amastigotes) contained detectable amounts of GFP (Figure 3A, 3C and 3D). In these cells, green fluorescence was strongly observed throughout the parasite body, including the epimastigote flagellum (Figure 3C and 3D). By contrast, no GFP fluorescence was detected in the non proliferative stages, i.e., metacyclic trypomastigotes (Figure 3C and 3D) and cell-derived trypomastigotes (Figure 3B).

**Figure 4:**
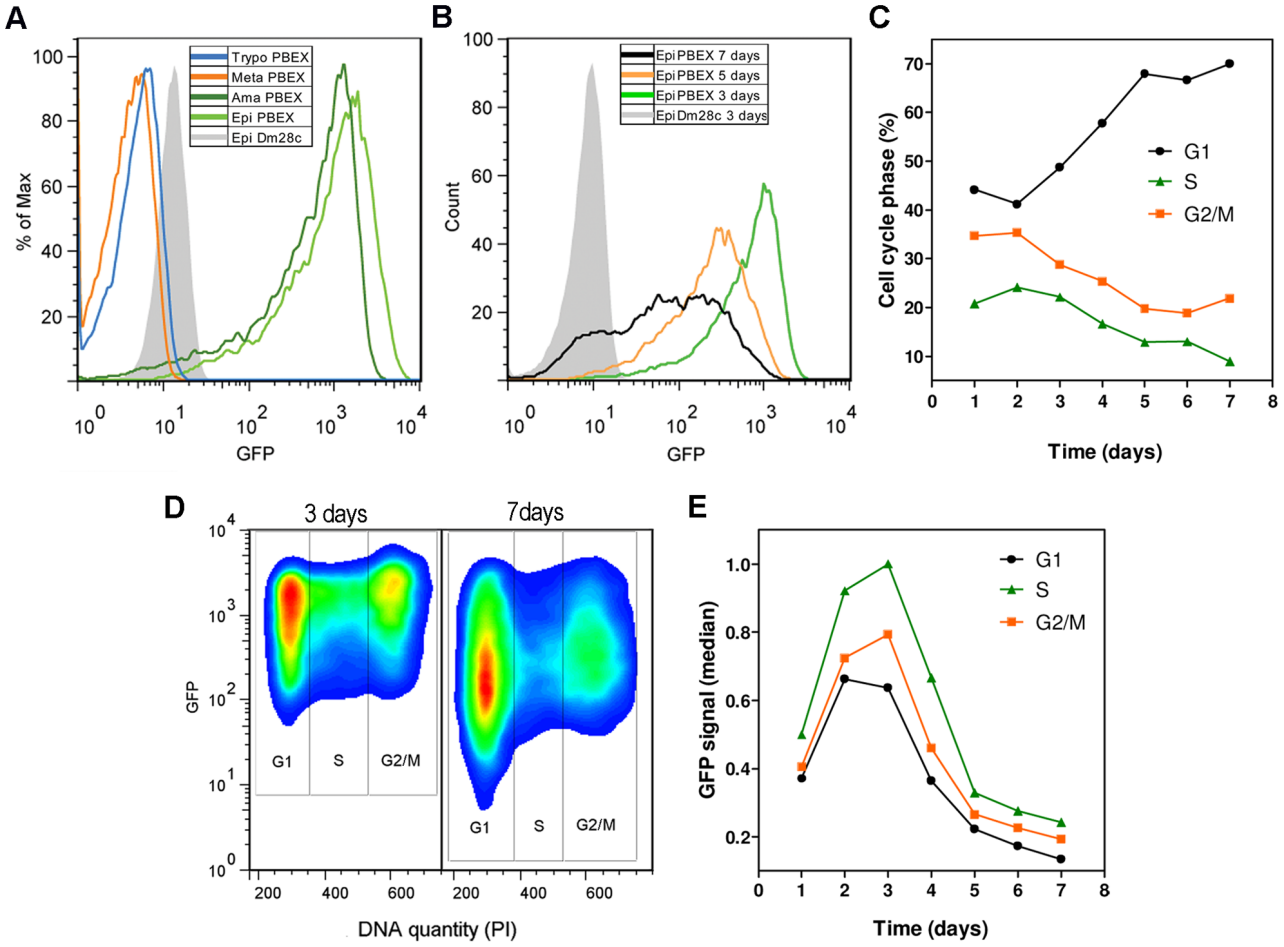
Flow cytometry analysis of GFP fluorescence in all major stages of the *T. cruzi* pBEX/GFP life cycle. (A) Overlay histograms of GFP fluorescence in various forms of *T. cruzi* (specified in the box). Note that only the replicative forms (epimastigotes and amastigotes) of the parasite display GFP fluorescence. % of Max is a normalization of the total number of events in the overlaid histograms; each sample is scaled to the percentage of its maximum signal. (B) Overlay histograms of GFP fluorescence after various numbers of days of epimastigote culture; exponential growth phase (3 days), stationary phase (5 days) and late stationary phase with the presence of metacyclic forms (7 days). Cell culture was initiated with 1×10^6^ cells ml^−1^ (as in Figure 2A). (C) Percentage of epimastigotes in each phase of the cell cycle (G1, S and G2/M), as determined by flow cytometric analysis of DNA content with PI staining, during epimastigote culture. (D) Flow cytometry density plot showing the correlation of GFP fluorescence with the cell cycle of epimastigotes of *T. cruzi* pBEX/GFP after 3 days and 7 days of culture (indicated above the plot). (E) Median GFP fluorescence in each phase of the cell cycle during the culture of pBEX/GFP epimastigotes. The intensity of GFP expression is higher in cultures displaying active replication of DNA (S phase) and is highly dependent on culture time. In the plots, wild-type epimastigotes (Dm28c) were used as a negative control for the normalization of GFP fluorescence level. The results shown in (C), (D) and (E) are representative of duplicate experiments with coefficients of variation of less than 5%.

For confirmation of the results of fluorescence microscopy experiments, we analyzed the GFP signal by flow cytometry. Amastigotes and epimastigotes were found to have high levels of GFP fluorescence, whereas the infective stages displayed no GFP fluorescence (Figure 4A).

Cell fluorescence level was correlated with the replicative state of the parasite. Exponentially growing epimastigotes had higher levels of fluorescence than parasites in the stationary growth phase (Figure 4B). After seven days of epimastigote culture, viable non fluorescent parasites, corresponding to differentiating epimastigotes and fully differentiated metacyclic forms (black line in Figure 4B) were observed. We then investigated the correlation of GFP fluorescence level with DNA replication by carrying out cell cycle analyses, in which total DNA was stained with propidium iodide. We found that GFP fluorescence (Figure 4C, D and E) was indeed strongly influenced by the percentage of dividing parasites (cells in the S and G2/M phases) in the culture (Figure 4C). As the culture aged and reached the stationary phase (fewer cells in the S and G2/M phases, Figure 4C), GFP signal intensity decreased (Figure 4D and 4E). Regardless of the growth phase of the parasite, GFP fluorescence (Figure 4E) was strongest in actively replicating cells (S phase), followed by cells in the G2/M and G1/G0 phases, which had lower median levels of GFP fluorescence.

We investigated whether recombination of the pBEX/GFP vector with chromosomes containing rDNA loci was indeed the main factor involved in the regulation of GFP expression during the parasite lifecycle, by analyzing four other clones of *T. cruzi* pBEX/GFP. All had similar levels of GFP fluorescence and were also fluorescent only at the epimastigote and amastigote stages (results similar to those shown in Figure 4A and 4B). Furthermore, the presence of G418 (at various doses) had no effect on GFP fluorescence intensity (data not shown). We analyzed the genomes of all these clones with the 18S, 24S and GFP probes after PFGE and found that GFP was integrated into a chromosome carrying rRNA genes in all cases (results similar to those shown in Figure 1B and 1C). These results are consistent with the integration of the construct into the parasite genome at rDNA loci. The observation of GFP fluorescence exclusively in replicative stages of the parasite (epimastigotes and amastigotes) in all five clones tested, is consistent with stage-specific gene expression based on Pol I transcriptional activity at rDNA loci during the *T. cruzi* life cycle (details in the Discussion). The mechanism of GFP regulation in pBEX/GFP clones is, therefore, probably an ideal tool for studying the regulation of gene expression at rDNA loci in this parasite.

The stronger GFP fluorescence signal in exponential-growth phase parasites and the limitation of fluorescence to replicative stages of *T. cruzi* may be accounted for by the higher rates of RNA metabolism in these cells. Indeed, replicative forms of *T. cruzi* have much higher levels of RNA polymerase activity, with transcription rates for both RNA pol I and II 10 times higher than those in infective forms [34]. Interestingly, it has recently been shown that transfection resulting in the stable insertion of the GFP or RFP gene into the tubulin locus of *T*. *cruzi* results in intense fluorescence in all parasite stages [23].

**Figure 5:**
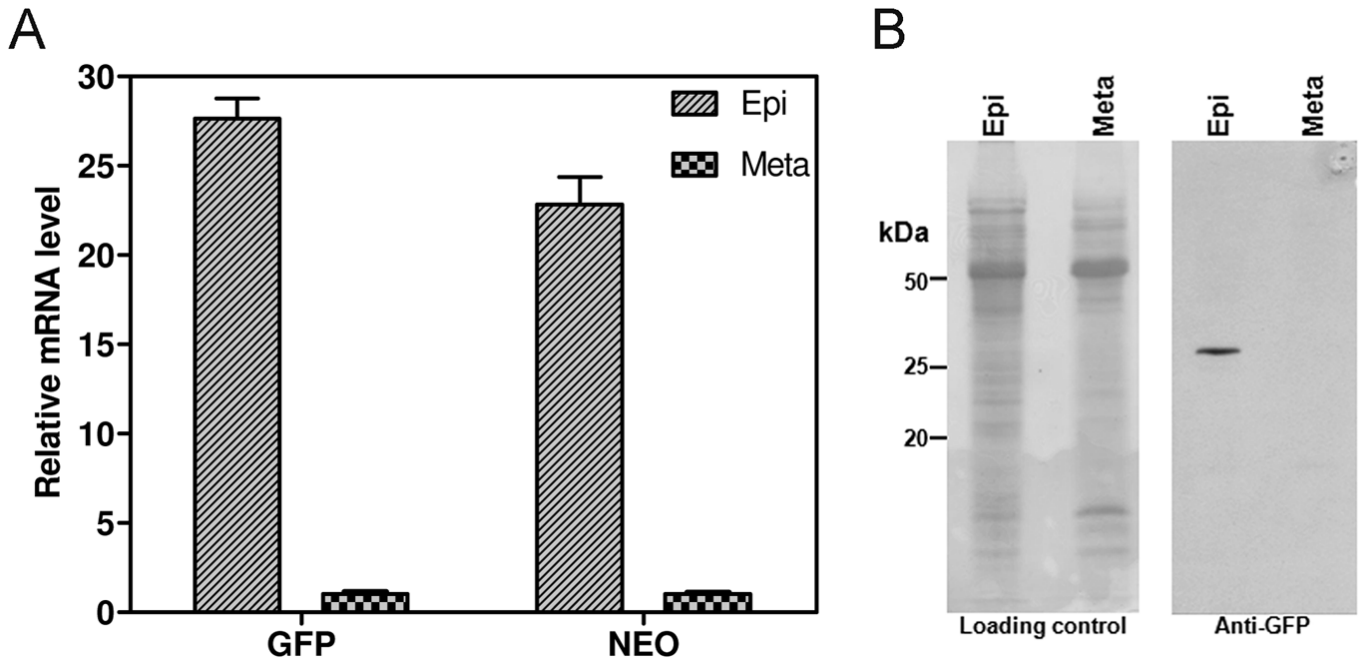
RT-qPCR and western-blot analysis of GFP expression. (A) mRNA levels for the *GFP* and *NEO* genes in epimastigotes and metacyclic trypomastigotes, as determined by RT-qPCR and shown as relative mRNA levels, with the metacyclic level fixed at 1. (B) Western blot of GFP in epimastigotes (Epi) and metacyclic trypomastigotes (Meta). The “Loading control” (left) is the Ponceau-stained membrane before incubation with anti-GFP antibody (right). Note the detection of GFP only in epimastigotes.

We then carried out RT-qPCR to determine whether GFP expression levels in the various parasite stages were correlated with GFP transcript levels. Epimastigotes and metacyclic trypomastigotes were used in these experiments to represent *T. cruzi* replicative and non proliferative stages, respectively. RT-qPCR showed that epimastigotes had about 28 times more GFP mRNA than metacyclic forms (Figure 5A). Similarly, RT-qPCR analysis showed that expression of the *NEO* gene, the resistance marker downstream from the GFP gene (Figure 1A), was also stronger (by a factor of about 23) in epimastigotes than in metacyclic forms (Figure 5A). Western-blot analysis of GFP levels demonstrated an absence of detectable levels of this protein in metacyclic forms (Figure 5B). Together, these results confirmed those of flow cytometry and fluorescence microscopy experiments, demonstrating the production of detectable amounts of GFP only in the replicative stages of the parasite.

### Correlation of GFP Fluorescence Intensity and Cell Number: Applications for Drug Screening Assays

**Figure 6:**
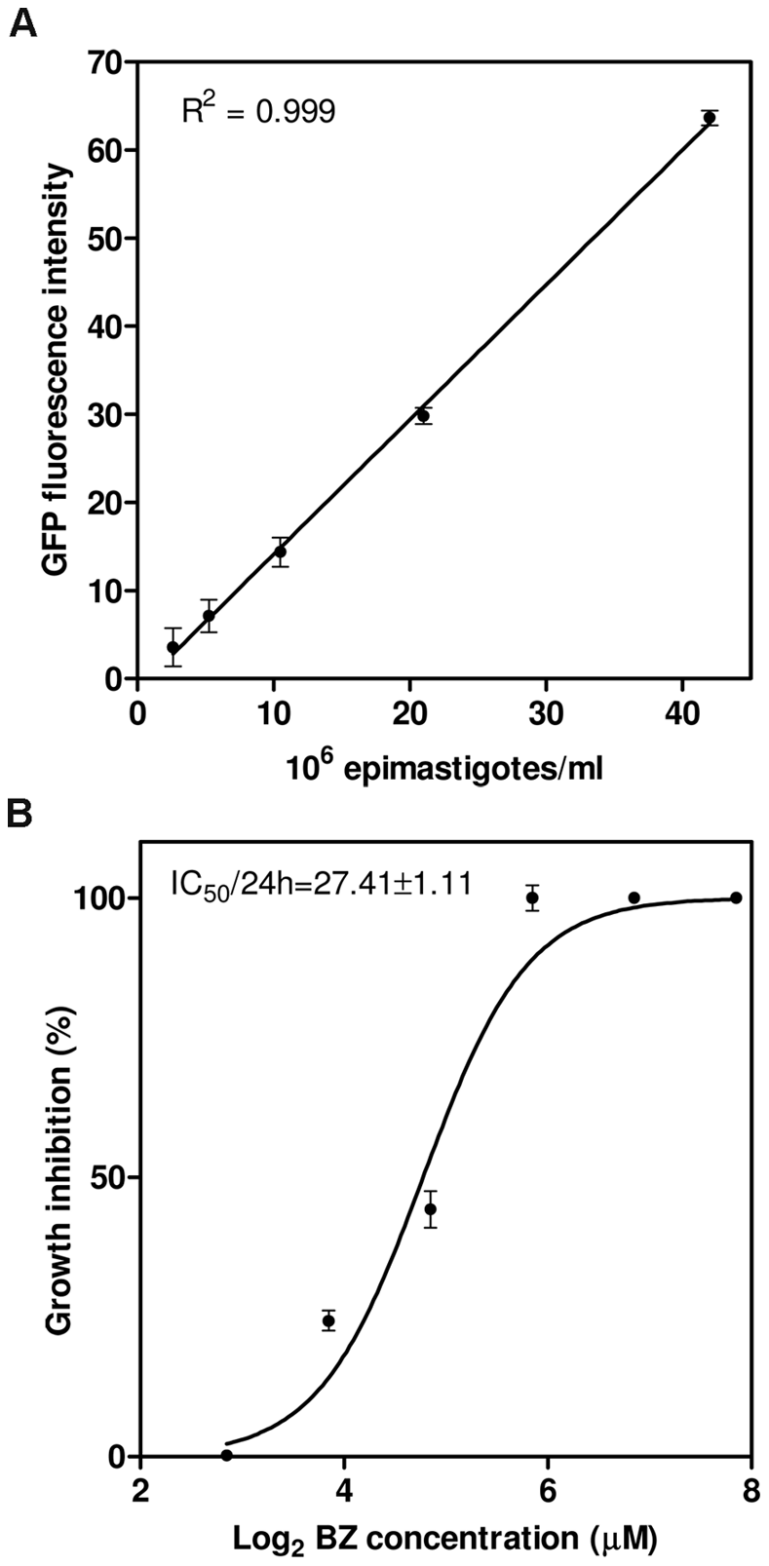
Fluorometric assay of parasite density. (A) Correlation between cell density and the GFP fluorescence intensity obtained with a microplate reader. Epimastigotes were diluted to known densities in LIT medium in triplicate before fluorescence determinations; the total cell number and fluorescence intensity were strongly linearly correlated (R^2^ = 0.999). (B) Calculation of the benznidazole inhibitory dose by the microplate fluorometric method. Each experimental point represents the mean and standard deviation for quintuplicate drug exposures. The IC_50_/24 h is indicated on the graph.

We investigated the use of the GFP-producing *T. cruzi* cell line described here for *in vitro* drug screening assays. We first analyzed the correlation between GFP fluorescence intensity and cell density in a microplate fluorescence reader, using serial dilutions of epimastigotes at mid-exponential growth phase. A linear relationship was found between parasite number and GFP fluorescence, with a linear correlation coefficient (R^2^) of 0.999 (Figure 6A). The fit of the linear regression was optimal for cell densities above 1×10^6^ parasites ml^−1^, whereas parasite fluorescence was overwhelmed by the autofluorescence of LIT medium below this threshold. As the cell density used for *T. cruzi* culture and drug tests usually exceeds 1×10^6^ cells ml^−1^, this method is potentially useful as a rapid and simple means of estimating parasite cell density.

We validated our drug screening method based on the fluorescence intensity of GFP-expressing epimastigotes, by calculating the IC_50_/24 h of benznidazole, the drug classically used to treat Chagas disease. For epimastigotes, we obtained an IC_50_/24 h of 27.41±1.11 µM (Figure 6B), which is within the range obtained for several other *T. cruzi* strains [35] and similar to the value obtained for the Dm28c strain [36]. This method could therefore improve the screening of anti-*T.cruzi* drugs in high-throughput systems, by reducing the manipulations in culture required for the calculation of inhibitory doses. It is also much simpler than the classical laborious microscopic counting methods and MTT assays for determining cell viability [19].

**Figure 7:**
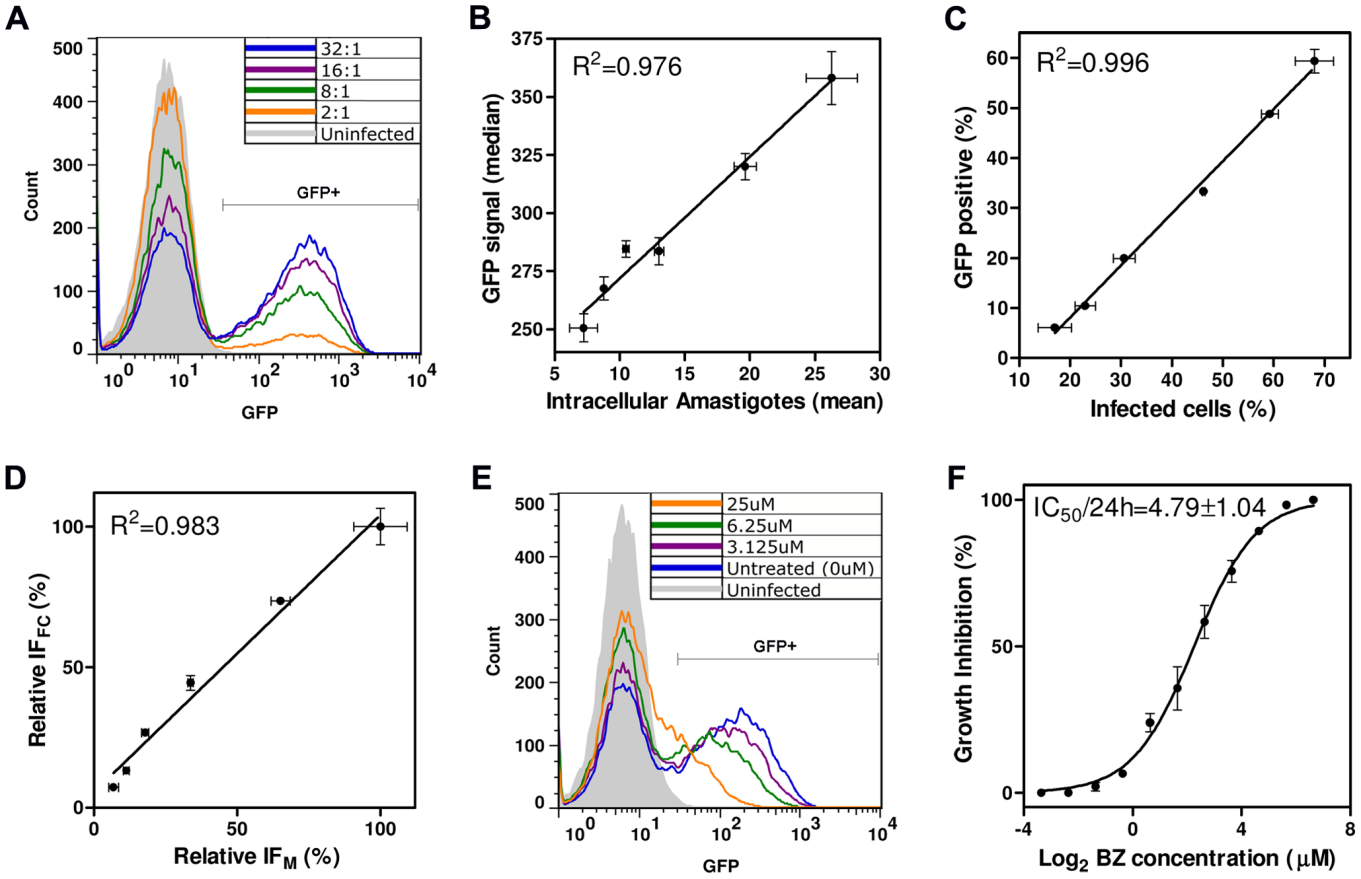
Flow cytometry assay for intracellular anti-amastigote activity. (A) Overlaid histograms of GFP fluorescence in Vero cells infected with various ratios of trypomastigotes/Vero cells (indicated within the graph) together with a non infected control. The GFP^+^ Vero cells were considered to be infected with amastigotes. (B) Correlation between the mean number of amastigotes per Vero cell (obtained by manual counting on Giemsa-stained smears) and the median GFP signal of GFP^+^ Vero cells (obtained in flow cytometry experiments). Each experimental point corresponds to Vero infection with a different ratio of trypomastigotes/Vero cells (as in (A)). Note that the results of the two methods of intracellular amastigote quantification display a strong linear correlation (R^2^ = 0.976). (C) Correlation between the % infected cells obtained by microscopy and flow cytometry (R^2^ = 0.996). (D) Comparison of the infection indices in Vero cells obtained by microscopy (IF_M_) and flow cytometry (IF_FC_), to estimate intracellular amastigote growth. Data are shown as a percentage of maximum growth. The results of the two methods are highly correlated (R^2^ = 0.983). For details of IF_M_ and IF_FC_ calculation, see Materials and Methods. (E) Overlaid histograms of infected Vero cells (10 trypomastigotes/Vero cell) treated with different doses of benznidazole (indicated within the graph) together with uninfected and untreated controls. (F) Calculation of the benznidazole IC_50_/24 h (indicated within the graph) by the flow cytometry method. Each experimental point represents the mean and standard deviation of duplicate drug exposures.

We then developed a flow cytometry assay to determine whether the number of intracellular amastigotes in infected Vero cells was correlated with the GFP fluorescence signal. We first compared the classic Giemsa staining method with the flow cytometric quantification of Vero cells infected with fluorescent amastigotes, using several different infection ratios (trypomastigote/host cell). Flow cytometry was found to be a simple and rapid method for determining the percentage of infected cells on the basis of the GFP fluorescence generated by intracellular amastigotes (Figure 7A). We also found that the median GFP signal intensity within the GFP^+^ population correlated well with the mean number of amastigotes per infected cell obtained by light microscopy analysis (linear correlation coefficient of 0.976, Figure 7B). Furthermore, the percentage of infected cells obtained by the two methods followed a linear correlation (R^2^ = 0.996, Figure 7C). However, the flow cytometry method systematically yielded a smaller percentage of infected cells (Figure 7C), probably due to the disruption of highly infected cells by trypsinization. However, multiplication of the percentage infected cells by the median GFP^+^ signal (IF_FC_) and comparison of the result obtained with the IF_M_ (percentage infected cells determined by microscopy multiplied by the mean number of amastigotes per infected cell), showed the infection indices of the two methods to be well correlated (R^2^ = 0.983). Flow cytometry is thus a robust method for quantifying intracellular amastigote growth, even when the number of amastigotes within cells is low.

We validated our flow cytometry assay for intracellular amastigote analysis, by applying it to BZ treatment (Figure 7E). We obtained an IC_50_/24 h of 4.79±1.04 µM (Figure 7F). This value is very close to that already obtained for intracellular amastigotes [37], indicating that this method could be used to screen for new anti-*T. cruzi* drugs affecting the clinically relevant amastigote stage. It is also possible to use other fluorescent probes in the same assay, such as fluorochromes for assessing cell viability, making it possible to incorporate the analysis of mammalian cell cytotoxicity into the same assay.

One possible drawback of this assay is related to the use of compounds likely to induce differentiation into non fluorescent forms (metacyclic or cell-derived trypomastigotes), potentially providing false estimates of growth inhibitory doses. However, this issue could easily be resolved by observing the cells by light microscopy before the analysis of growth inhibition.

## Discussion

Our understanding of many aspects of the pathogenesis and progression of Chagas disease remains incomplete. Moreover, current treatments for this disease are only partly effective and are toxic, and there is no vaccine. There is therefore a need to develop tools providing better information about the biology of the parasite with a view to developing new strategies for the prevention and treatment of this disease.

We describe here the construction of a fluorescent *T. cruzi* strain in which the GFP gene is integrated into the 18S ribosomal locus. Unlike other fluorescent *T. cruzi* cell lines [22], [23], this GFP-producing *T. cruzi* clone displays detectable levels of fluorescence only during replicative stages. Our RT-qPCR results indicated that GFP and NEO mRNA levels were about 25 times higher in epimastigotes than in metacyclic trypomastigotes. We speculate that GFP expression is controlled by the endogenous ribosomal promoter, which may be inactive in the non replicative stages of the parasite due to epigenetic events involving chromatin remodeling and compaction. Accordingly, transcription is highly active in epimastigote forms and decreases significantly in fully differentiated metacyclic trypomastigote forms [34], [38]. This differentiation process is also accompanied by an extensive reorganization of the nucleus, with large amounts of heterochromatin found throughout the elongated nucleus of metacyclic forms, and dispersion of the contents of the nucleolus [34]. GFP expression is probably also controlled by differences in protein stability between the various parasite stages [39].

Two widely used episomal expression vectors in *T. cruzi*, the pRIBOTEX [11] and pTREX [14], [15] systems, harbor a ribosomal promoter upstream from the cloning site used for the transgene. The presence of this exogenous ribosomal promoter improves gene expression and leads to the integration of these vectors into the parasite genome at the ribosomal locus [14], despite both ends of the linearized pTREX vector being homologous to the *gapdh* locus of *T. cruzi*. Recombination occurs through an unknown illegitimate mechanism [13]. Interestingly, it has recently been shown that the transfection of *T. cruzi* with pTREX harboring tandem tomato fluorescent protein genes (pTREX-Neo-tdTomato) [40] resulted in transfectant parasites with bright red fluorescence distributed throughout the cell, at all stages of the life cycle. Another recent study showed that the insertion of fluorescent protein (GFP and RFP) genes into the tubulin locus (transcribed by Pol II) by homologous recombination [23] also resulted in parasites with high levels of fluorescence throughout their life cycle. By contrast, the *T. cruzi*-GFP cell line described here displayed detectable levels of fluorescence only during replicative stages. The pBEX plasmid, unlike pTREX, does not carry a rRNA promoter and contains segments of the 18S rRNA gene flanking the GFP/NEO cassette, sufficient to target this cassette to the 18S locus by homologous recombination. Thus, pBEX/GFP *T. cruzi* expresses the transgene via Pol I-mediated transcription under the control of the native rRNA promoter. This characteristic feature may account for the expression of GFP in replicative stages of the parasite only. The mechanism of rRNA/GFP regulation in this clone is currently being investigated and may prove a useful tools for studies of the regulation of gene expression at the rDNA loci during the *T. cruzi* life cycle.

The GFP gene has also recently been integrated into the 18S rDNA locus of *L. mexicana* [33], *L. major* and *L. donovani* [20], [41], by homologous recombination. Interestingly, in these species, GFP is produced in large amounts throughout the life cycle of the parasite, by contrast to our findings for *T. cruzi*. Promastigotes in the exponential and stationary growth phases also had similar levels of GFP fluorescence [20]. These findings may reflect biological differences between the two parasites, as both *Leishmania* stages (promastigotes and amastigotes) are active replicative forms, whereas transcription levels in non replicative infective stages of *T. cruzi* are lower than in epimastigotes [34].

Drug-based treatments are currently the only option for most parasitic diseases. The construction of GFP-expressing parasites has made it possible to develop simple and reliable drug-screening methods for the search for new molecules effective against trypanosomatid pathogens (reviewed in [16]). FP-based techniques provide a real-time analysis of parasite infection without the need for additional preparation.

The fluorescence signal in pBEX/GFP *T. cruzi* was strongly correlated with the total number of parasites in cultures, providing a rapid and simple means of estimating the concentrations of drugs inhibiting parasite growth in high-throughput screening assays, as already demonstrated for *L. amazonensis* [42], [43] and *T. cruzi* [40]. We used the fluorescent *T. cruzi* clone described here as a tool for determining the inhibitory doses of benznidazole in epimastigotes, by microplate fluorometric assays, and in intracellular amastigotes, by flow cytometry. As far as we know, this is the first demonstration of a FACS-based anti-amastigote drug screening assay for *T. cruzi*. As already reported for *Leishmania* species [44], [45], this method requires significantly less time for preparation and analysis than manual counting under a light microscope.

Transgenic parasites expressing reporter genes also constitute a useful tool for visualizing the parasite infection directly in the host with specialized imaging systems (reviewed in [16], [46]). Recent studies in *Leishmania* [20], [47] and *T. cruzi* [40] have demonstrated the potential of fluorescent protein-producing parasites for the dynamic follow-up of parasite propagation in infected animals. The *T. cruzi* cell line described here could thus be used to monitor host infection directly, through the use of fluorescence imaging systems, with exciting implications for *in vivo* drug-screening assays and vaccine development. As only the replicative stages are fluorescent, a more refined analysis of the rate of proliferation in the host is possible. Furthermore, FACS technology can be used to obtain fluorescent amastigotes or epimastigotes directly from infected mammal tissues or insect vectors, respectively.

A recurrent problem in the study of *T. cruzi* is the isolation of trypomastigotes and amastigotes from infected mammalian cell cultures. Various protocols have been described in recent decades [48], [49], [50], but all are time-consuming and suffer from low efficiency and an excessive need for parasite manipulation. The *T. cruzi* strain described here displays differential GFP expression between the infective and replicative forms. Thus, fluorescence-activated cell sorting (FACS) can be used to separate trypomastigotes from amastigotes. In preliminary FACS experiments, we obtained highly pure preparations of the different forms after cell sorting, minimizing contamination problems for the biological analysis of specific stages.

In conclusion, the stably GFP-transfected *T. cruzi* described here is a useful tool for *in vitro* and *in vivo* analysis of parasite biology. The restriction of GFP fluorescence to replicative stages is potentially useful in several fields, including studies of gene expression regulation, host-parasite interaction, drug screening and vaccine development.
